# A neurobiological enquiry into the origins of our experience of the sublime and beautiful

**DOI:** 10.3389/fnhum.2014.00891

**Published:** 2014-11-11

**Authors:** Tomohiro Ishizu, Semir Zeki

**Affiliations:** Wellcome Laboratory of Neurobiology, Division of Biosciences, Department of Cell and Developmental Biology, University College LondonLondon, UK

**Keywords:** beauty, emotion, fMRI, neuroesthetics, sublimity

## Abstract

Philosophies of aesthetics have posited that experience of the sublime—commonly but not exclusively derived from scenes of natural grandeur—is distinct from that of beauty and is a counterpoint to it. We wanted to chart the pattern of brain activity which correlates with the declared intensity of experience of the sublime, and to learn whether it differs from the pattern that correlates with the experience of beauty, reported in our previous studies (e.g., Ishizu and Zeki, 2011). 21 subjects participated in a functional magnetic resonance imaging experiment. Prior to the experiment, they viewed pictures of landscapes, which they rated on a scale of 1–5, with 5 being the most sublime and 1 being the least. This allowed us to select, for each subject, five sets of stimuli—from ones experienced as very sublime to those experienced as not at all sublime—which subjects viewed and re-rated in the scanner while their brain activity was imaged. The results revealed a distinctly different pattern of brain activity from that obtained with the experience of beauty, with none of the areas active with the latter experience also active during experience of the sublime. Sublime and beautiful experiences thus appear to engage separate and distinct brain systems.

## Introduction

When Edmund Burke (1757) published *A Philosophical Enquiry into the Origin of Our Ideas of the Sublime and Beautiful*, of which our title is an imitation, he was removed by centuries from early speculations on the origins of the sublime and its experience. History traces those origins to Longinus, a Greek philologist of doubtful existence also known as pseudo Longinus, who thought of the sublime as “a certain loftiness and excellence of language” (Havel, 2006). While acknowledging that “words have as considerable a share in exciting ideas of beauty and of the sublime” (Burke, 1757, 5: I), for Burke and others during the Enlightenment, the trigger for the experience of the sublime shifted toward a more sensory base. Now the sublime was found in the experience of Nature and its uncontrollable forces: the untameable power of oceans, the grandeur of mountains, the terror of erupting volcanoes, and the vastness of deserts became some of its principal sources. Yet a common theme across the centuries dividing Longinus from Burke was that the source of the experience was traceable to the workings of the mind, rather than the objects of Nature exclusively. Critical to that experience was the involvement of the imagination and the process of completion, since it posited a profound interaction between the apprehended object and its completion by the recipient; the sublimity lay beyond the power of the senses and therefore recruited the power of the imagination. Longinus wrote that “sublime thoughts belong properly to the loftiest minds.” Kant (1790/1986), somewhat more emphatically, stated that sublimity “is not contained in anything in nature, but only in our mind” (§28, 264), giving an example from mathematics where, “to be able to think the infinite as a whole indicates a mental power that surpasses any standard of sense” (§26, 255). Here, then, was a critical difference from the experience of beauty, for which there was a more equitable division, especially in the Platonic tradition, between sensory experience and the characteristics of the apprehended object. These objects and Ideas had, for Plato, an existence independent of humans even though they could only be accessed through a thought process.

A greater emphasis on the imagination is not the sole characteristic separating the experience of the beautiful from that of the sublime. While beauty came to be associated with feelings of pleasure, reward, and satisfaction, the sublime was associated with awe, fear and terror, because “Whatever therefore is terrible, with regard to sight, is sublime too” (Burke, 1757, 2: II). But, significantly, these feelings were read into scenes from a safe distance. The sublime was associated, above all, with the obscure and the uncertain, with “some sort of approach toward infinity” (Burke, 1757, 2: IV).

These differences, listed by Burke, Kant, and Schopenhauer among others, are impressive and compelling. In pursuing our studies of the neurobiology of aesthetic experiences, we therefore thought it interesting to enquire whether they are also mirrored in the pattern of brain activity that correlates with the two experiences. We naturally approached our experimental project with diffidence. However, compelling the differences between the two experiences may be at the extremes, there remain many experiences, of which the *Pietà* of Michelangelo at St Peter's Basilica is a supreme example, which are easy to characterize as both beautiful and sublime. Indeed, Schopenhauer (1844, I: 289) resorted to giving different categories of the sublime, in terms of intensity of experience. Acknowledging these difficulties, we tailored our experiment so that each subject characterized the intensity of the experience of the sublime individually. Given the dominance accorded by philosophers of aesthetics to natural scenes in the experience of the sublime, we restricted ourselves to studying the experience of the sublime when aroused by such scenes. We then undertook a parametric imaging study of the strength of activity in the implicated brain areas, in relation to the declared intensity of the experience of the sublime. In light of the distinctions made in the philosophy of aesthetics between the experience of the beautiful and that of the sublime, we hypothesized that there would be a significant difference in the pattern of activation that correlates with the two experiences.

## Materials and methods

### Participants

Twenty-one healthy right-handed volunteers (11 males; 10 females; mean age, 26.6 years) coming from different cultures and ethnic backgrounds (3 Chinese, 4 Indian, 6 Japanese, 2 Pakistani, and 6 West Europeans) participated. All had normal or corrected-to-normal vision, and none had a history of neurological or psychiatric disorder. Written informed consent was obtained from all participants. The study was approved by the Ethics Committee of University College London. All data were anonymized.

### Preliminary behavioral testing and post-scanning ratings

Prior to the scanning sessions, psychophysical tests were conducted to select stimuli which were subsequently used in the scanning sessions; this allowed subjects to classify the visual stimuli into five groups according to the intensity with which they experienced them- 5 corresponding to “very sublime,” 1 to “not at all sublime” and the others falling in between. These ratings allowed us to balance the sequence of stimuli for each participant in order to achieve an even distribution of visual stimuli of all 5 ratings during the scanning sessions. During a first visit to the laboratory, between 2–5 days prior to scanning, each subject was instructed about the experiment and rated the stimuli as described above. Each viewed 232 photographs of natural scenes from the National Geographic Magazine available on the internet; these included pictures of mountains, falls, forests, volcanoes, tornadoes, ocean waves, glaciers, clouds, and deserts, that is to say the type of stimuli which have been considered in literature to evoke feelings of the sublime. Stimuli remained on the screen until participants responded, after which an inter-trial interval of 1 s followed. Based on the responses of each participant during the preliminary psychophysical testing, we selected—for each participant—35 stimuli corresponding to each of the 5 categories, making a total of 175 stimuli which each participant viewed in the scanner. Immediately after scanning, each participant was asked to report their affective feelings with regards to the “beauty,” “pleasantness,” and “scale” of each stimulus that they had viewed in the scanner, on a scale of 1 to 5 (ugly to beautiful for “beauty,” fearful to pleasant for “pleasantness,” and 5 to 1 (small to grand for “scale”). We chose a reverse rating for scale, because it has been posited in philosophies of aesthetics that large scales (sizes) are more characteristic of the sublime, while small ones are more characteristic of the beautiful (Burke, 1757, 3: XXVII).

### Stimuli

Stimuli were generated with Cogent 2000 (http://www.vislab.ucl.ac.uk/cogent_2000.php) running in MATLAB (MathWorks, Natick, MA, USA). They were back-projected onto a screen, by use of an LCD projector, through an angled mirror. The resolution of the screen was 1400 × 1050 pixels; the height of each stimulus was 19°, and the width varied.

The session began with subjects viewing a flat black screen for 20 s to allow for T1 equilibration effects to subside (the corresponding first six brain volumes were discarded). After this 20 s blank period, an instruction about the sublimity judgment appeared on the screen, to inform participants that a session had started. A fixation point was then presented at the center of the screen for 1 s against a black background, following which visual stimuli were presented in a pseudorandom order for 5 s. This was followed by an interval of 5–7 s, during which the participants responded.

Following each stimulus presentation, participants were asked to rate them on a five Likert scale as in the preliminary testing, by pressing one of five buttons with their right five fingers. The response period lasted 5–7 s and participants could make their rating at any time during that period. The session ended with a blank period of 20 s, during which the scanner continued to acquire blood-oxygen-level dependent (BOLD) signals. The stimuli were presented in 7 sessions. Each session consisted of 22 stimuli, presented in pseudorandom order, with a 20 s resting period between first and last 11 trials, during which participants were instructed not to close their eyes. Prior to the scanning, participants had a short practice session with different visual stimuli to those used in the scanning session.

### Functional magnetic resonance imaging (fMRI) scanning

Scanning data were acquired in a 3-T Siemens Magnetom Trio magnetic resonance imaging scanner (Siemens, Erlangen, Germany) fitted with a 12-channel head-coil. An echo-planar imaging (EPI) sequence was applied for functional scans to obtain BOLD signals (echo time, 30 ms; repeat time, 3.36 s), using 48 slices to cover the whole brain. The voxel resolution was 3 × 3-mm in-plane resolution, with a 2 mm slice thickness and 1-mm inter-slice gap. Magnetic resonance imaging signal losses in the orbitofrontal cortex (OFC) and amygdala were reduced by applying a z shim gradient moment and slice tilt (Weiskopf et al., 2006). T1-weighted anatomical images were acquired at the end of experimental sessions for each subject (176 slices; resolution, 1 × 1 × 1 mm; echo time, 2.48 ms; repeat time, 7.92 ms). Field maps were also acquired with Siemens standard gradient-echo field map sequence to correct geometric distortion of EPI images (Hutton et al., 2002). We also recorded the heart and respiration rates for each subject.

### fMRI data analysis

All data were analyzed with SPM8 (Statistical Parametric Mapping, http://www.fil.ion.ucl.ac.uk/spm/software/spm8/). The EPI images for each subject were realigned and normalized into Montreal Neurological Institute (MNI) space, smoothed with a Gaussian smoothing kernel of 9 × 9 × 9 mm, and filtered with a high-pass cut-off (128 s) to remove drift terms. The stimulus for each subject was modeled as a set of regressors in a general linear model first-level (within subject) analysis. The stimulus was a block design, and boxcar functions were used to define stimulus functions; these modeled the onsets and durations of the visual stimuli. Head movement parameters calculated from the realignment pre-processing step, physiological recordings, and the response periods were included as regressors of no interest. Stimulus functions were convolved with a canonical haemodynamic response function.

To analyze the data for parametric modulation according to intensity of experience, sublimity rated stimuli were used as regressors, with sublimity rating as the parametric modulator. Ratings were coded as 1, 2, 3, 4, and 5 for “not at all sublime” to “very sublime,” and a 1st order polynomial expansion was included. Contrast images for visual presentations and sublimity ratings were taken to second-level (random effects) analysis to produce summary statistical *t*-maps at the group level. In order to study de-activations produced by viewing the stimuli, we classified the latter as follows: stimuli given ratings of 1–2 as “Not sublime,” 3 as “Mid sublime,” and 4–5 as “Very sublime,” which allowed us to examine the de-activations produced by these three groupings relative to baseline. We carried out a categorical analysis of sublime rating, by coding contrasts for Very sublime, Mid sublime, Not at all sublime vs. Baseline for each subject at the first-level and taking these to a second-level, random effects, analysis.

We report cluster level activations that were significant at *p* < 0.05 FWE (family wise error) corrected, although some of these were also significant at the voxel level.

## Results

### Behavioral data

The percentage distribution of rating during the scanning experiment averaged over all participants for each of the five ratings of sublimity is 17.3% for 1 (*SD* = 7.81), 19.3% for 2 (*SD* = 9.28), 20.7% for 3 (*SD* = 7.21), 23.3% for 4 (*SD* = 9.00), and 19.5% for 5 (*SD* = 10.32). Thought not perfect, the distribution nevertheless provided a reasonable ratio between categories for the analyses.

**Figure 1:**
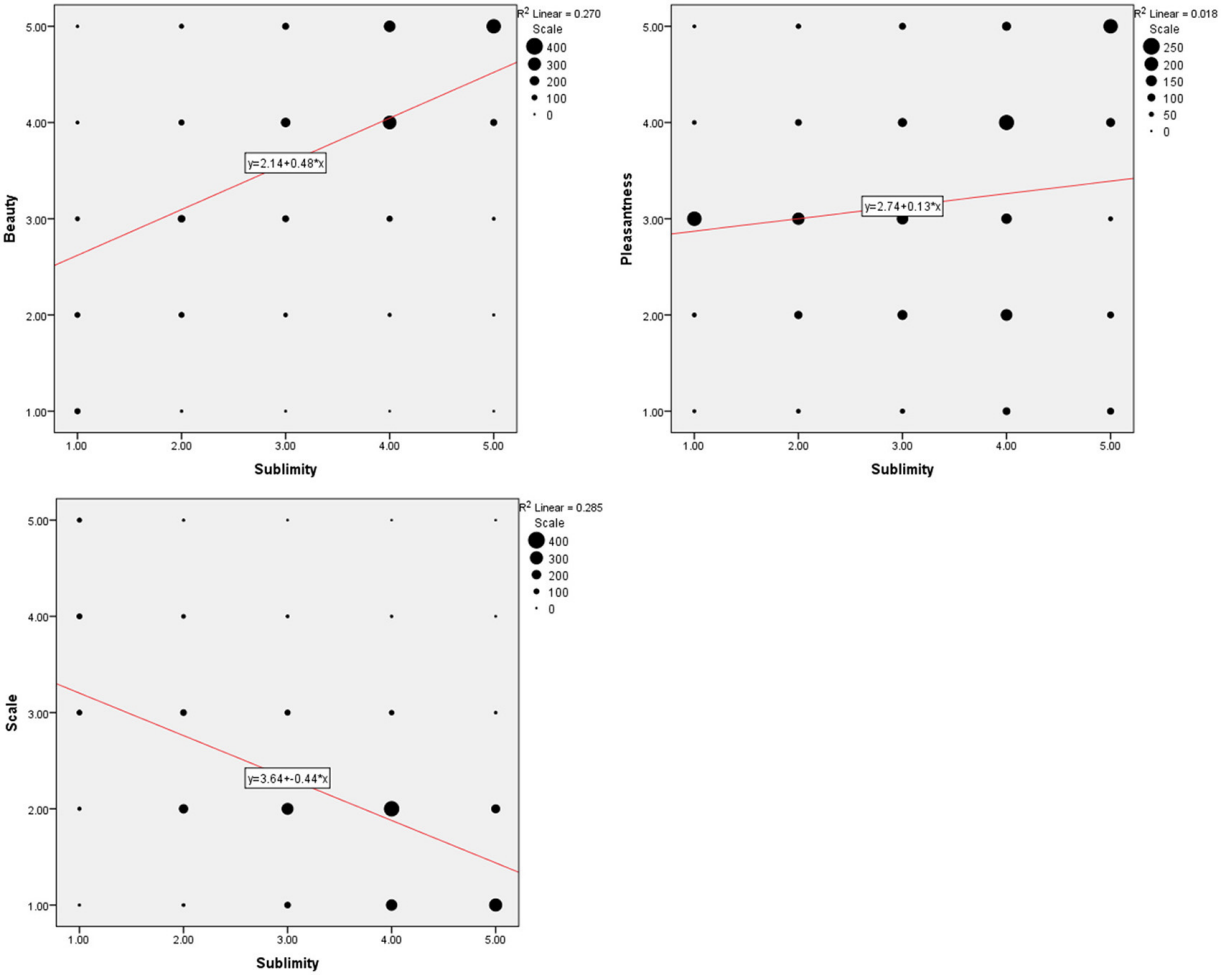
**Behavioral data summed over 21 subjects**. **(Upper left)** Frequency distribution of sublimity rating over post-scan beauty rating, **(upper right)** over post-scan pleasantness rating, **(bottom)** over post-scan scale rating, with a fitted linear regression. Size of each circle is proportional to the number for that rating.

Rating according to experienced sublimity carries with it certain confounds, since “In the infinite variety of natural combinations we must expect to find the qualities of things the most remote imaginable from each other united in the same object” (Burke, 1757, 3: XXVII). Chief among these is beauty, since stimuli experienced as sublime can also be experienced as beautiful or sometimes even ugly. In addition, sublimity often has fear and grandeur (magnitude or scale) attached to it. We therefore thought it wise to ask participants, after the scanning sessions, to rate the stimuli that they had viewed in the scanner along the three axes of beautiful—ugly, pleasant—unpleasant (fearful) and small—grand, each on a scale of 1 to 5 (but note that, as mentioned under Methods, we used a reverse rating for scale, compared to those sublime, beautiful, and pleasant). The frequency distribution of post-scan ratings for experiences besides sublimity (that is, those of beauty, pleasantness, and scale), produced by viewing the same stimuli as the ones in the scanner, are presented in Figure 1 and Table 1. Correlation analyses between ratings according to sublimity and each of the three post-scan ratings (Table 1) shows that sublimity ratings correlated positively with beauty (Pearson correlation, *r* = 0.520, *p* < 0.001, 2-tailed), weakly with pleasantness ratings (*r* = 0.136, *p* < 0.001), and had a significant negative correlation with scale (*r* = −0.534, *p* < 0.001).

**Table 1:** **The results of correlation analyses derived from the behavioral data over 21 subjects**.

		**Sublime**	**Beautiful**	**Pleasant**	**Scale**
Sublime	Pearson Correlation	1	0.520^**^	0.136^**^	−0.534^**^
	Sig. (2-tailed)		0.000	0.000	0.000
	*N*	2618	2618	2618	2618
Beautiful	Pearson Correlation	0.520^**^	1	0.452^**^	−0.545^**^
	Sig. (2-tailed)	0.000		0.000	0.000
	*N*	2618	2618	2618	2618
Pleasant	Pearson Correlation	0.136^**^	0.452^**^	1	−0.174^**^
	Sig. (2-tailed)	0.000	0.000		0.000
	*N*	2618	2618	2618	2618
Scale	Pearson Correlation	−0.534^**^	−0.545^**^	−0.174^**^	1
	Sig. (2-tailed)	0.000	0.000	0.000	
	*N*	2618	2618	2618	2618

** *p < 0.001*.

### Brain activations correlating with experience of the sublime

**Figure 2:**
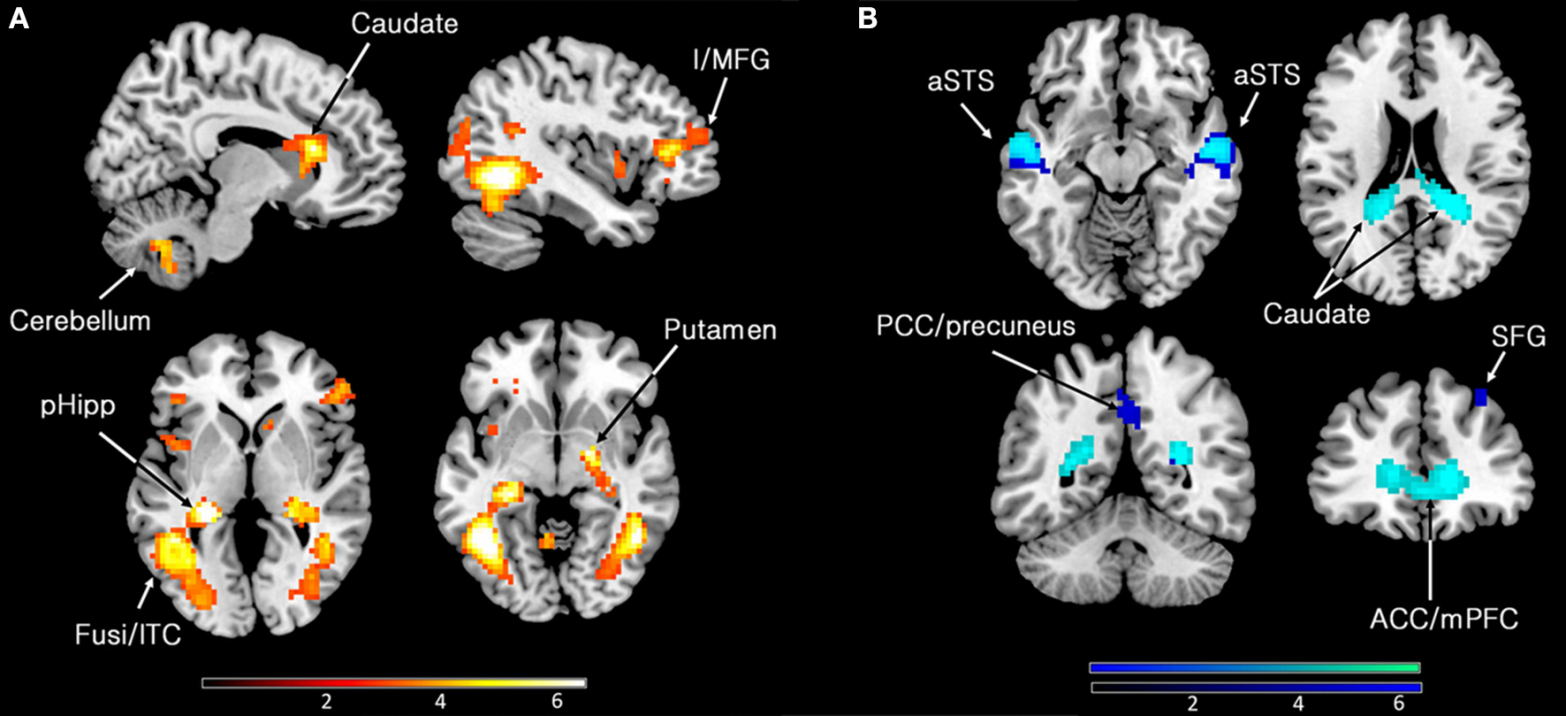
**(A)** Parametric activations with sublimity. Statistical parametric maps rendered onto canonical anatomical sections showing *t*-statistics. Random effects analysis with 21 participants. Display threshold at *p* < 0.001 (uncorrected). I/MFG, inferior/middle frontal gyrus (−39 35 10); pHipp, posterior hippocampus (−24 −31 1); Fusi/ITC, fusiform gyrus/inferior temporal cortex (48 −55 −8, −45 −58 −5); caudate (head) (9 23 16); cerebellum (9 −64 −44). **(B)** Sites deactivated during the experience of sublimity relative to baseline activity. Display threshold at *p* < 0.05 (corrected). Light blue indicates common deactivations for all contrasts, very sublime < baseline, mid sublime < baseline, and not sublime < baseline, and dark blue indicates sites deactivated uniquely during the presentation of the stimuli experienced as “very sublime.” aSTS, anterior superior temporal sulcus (54 −10 −8/−51 −16 −8); SFG, superior frontal gyrus (30 32 55); ACC/mPFC, anterior cingulate cortex/medial prefrontal cortex; (9 62 1); PCC, posterior cingulate cortex (0 −52 40); caudate (tail) (−18 −46 22).

Our primary interest was to determine the brain activity that correlates with the declared intensity of experiences qualified as sublime. A number of brain areas, cortical and subcortical, were found to have been parametrically active when sublime ratings were used as modulators (see Table 2 and Figure 2). The cortical areas were the inferior temporal cortex (encroaching upon fusiform gyrus and the lateral occipital complex (LOC), the posterior hippocampus, and the inferior/middle frontal gyri. The sub-cortical cortical areas were the basal ganglia (head of caudate and putamen). There was, in addition, prominent involvement of the cerebellum.

**Table 2:** **Location, MNI coordinates, cluster size and values for the activations produced by the parametric modulation with sublimity ratings and deactivations produced by the contrasts sublime < baseline**.

**Activations**		**Cluster**			**Peak**						
	**L/R**	***p*(FEW)**	**kE**	***p*(unc)**	***p*(FWE)**	***T***	***Z***	***p*(unc)**	***X***	***Y***	***Z***
Posterior hippocampus	L	0.000	1369	0.000	0.000	11.97	6.51	0.000	−24	−31	1
Inferior temporal cortex/fusiform gyrus	R				0.000	8.98	5.69	0.000	−45	−58	−5
					0.060	5.98	4.52	0.000	−30	−22	28
Inferior termporal cortex/fusiform gyrus	L	0.000	1648	0.000	0.000	9.14	5.74	0.000	48	−55	−8
Caudate(head)	R				0.002	7.96	5.35	0.000	9	23	16
Putamen	R				0.003	7.72	5.26	0.000	18	−7	−5
Cerebellum	L	0.001	272	0.000	0.005	7.40	5.14	0.000	−30	−61	−47
Celebellum	L				0.031	6.36	4.70	0.000	−21	−70	−44
					0.074	5.86	4.46	0.000	12	−55	−50
Inferior/midd1e fronta1 gyrus	L	0.015	146	0.003	0.038	6.24	4.64	0.000	−39	35	10
					0.111	5.62	4.34	0.000	−54	35	13
					0.660	4.39	3.65	0.000	−42	50	19
Cerebellum	R	0.057	97	0.013	0.045	6.14	4.60	0.000	27	−64	−44
**Deactivations**		**Cluster**			**Peak**						
		***p*(FWE)**	**kE**	**p(unc)**	***p*(FEW)**	***T***	***Z***	***p*(unc)**	***X***	***Y***	***Z***
Caudate (tall)	R	0.000	235	0.000	0.000	10.49	65535	0.000	21	−43	19
					0.004	5.60	5.00	0.000	33	−43	4
					0.007	5.42	4.87	0.000	27	−25	31
Anterior cingulate cortex	R	0.000	874	0.000	0.000	9.49	7.39	0.000	12	41	4
					0.000	8.16	6.67	0.000	15	32	7
Anterior ungulate cortex/medial prefrontal cortex	R				0.000	6.85	5.87	0.000	9	62	1
Anterior superio tempora1 sulcus	R	0.000	236	0.000	0.000	8.31	6.75	0.000	54	−10	−8
					0.001	5.99	5.28	0.000	63	−25	−8
					0.001	5.97	5.26	0.000	54	14	−29
Anterior superio tempora1 cortex	L	0.000	148	0.000	0.000	7.53	6.29	0.000	−51	−16	−8
					0.000	7.09	6.02	0.000	−54	−**7**	−11
Cerebellum	L	0.000	103	0.000	0.000	7.46	6.25	0.000	−24	−82	−32
		0.000	167	0.000	0.000	7.19	6.08	0.000	−27	−43	10
Caudate (tail)	L				0.000	6.83	5.85	0.000	−18	−46	22
					0.000	6.25	5.47	0.000	−12	−34	22
Cerebellum	R	0.000	78	0.002	0.000	7.10	6.03	0.000	21	−76	−35
					0.000	6.98	5.95	0.000	18	−85	−32
Caudate (body)	L	0.000	66	0.003	0.000	6.34	5.53	0.000	−24	−13	34
					0.001	6.11	5.37	0.000	−21	−4	31
					0.005	5.57	4.98	0.000	−24	−25	34
Caudate (body)	R	0.002	27	0.042	0.009	5.34	4.81	0.000	21	−1	28
					0.012	5.28	4.76	0.000	21	8	25
					0.031	4.96	4.53	0.000	21	−13	31
**DEACTIVATIONS UNIQUE TO VERY SUBLIME**											
Posterior cingulate cortex/precuenus	−	0.001	42	0.014	0.003	5.71	5.08	0.000	0	−52	40
Superior frontal gyrus	R	0.006	15	0.117	0.06	5.51	4.94	0.000	30	32	55

### Deactivations

Next, we investigated which brain areas were deactivated relative to baseline, using the following contrasts: very sublime < baseline, mid sublime < baseline, and not sublime < baseline. The results, summarized in Table 2 and Figure 2, show that all contrasts give significant deactivations in a number of areas. These include the anterior part of cingulate cortex/medial prefrontal cortex, anterior superior temporal sulcus, parts of the cerebellum, and the caudate (tail and body). The significant deactivations in posterior cingulate cortex/precuneus and superior frontal gyrus were only obtained with stimuli rated as very sublime.

**Figure 3:**
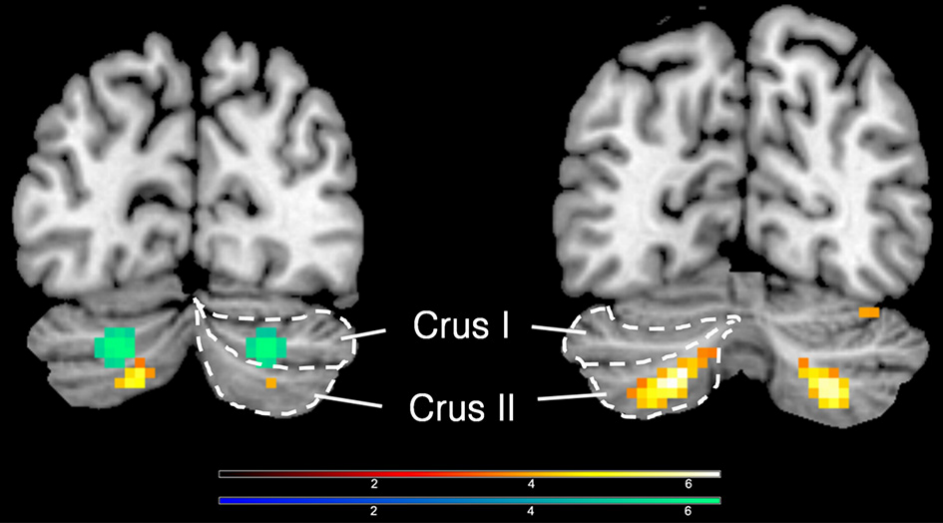
**Activations and deactivations in the cerebellum; yellow shows activations (−30 −61 −47/27 −64 −44) and green shows deactivations (−24 −82 −32/21 −76 −35), when an inclusive mask covering the whole cerebellum was applied**.

Deactivations in the cerebellum could be dissociated from the activations in it (peak voxels for deactivation, −24 −82 −32/21 −76 −35; for activation, −30 −61 −47/27 −64 −44, Figure 3).

### Comparison of brain areas whose activity correlates with the experience of the sublime and that of the beautiful

**Figure 4:**
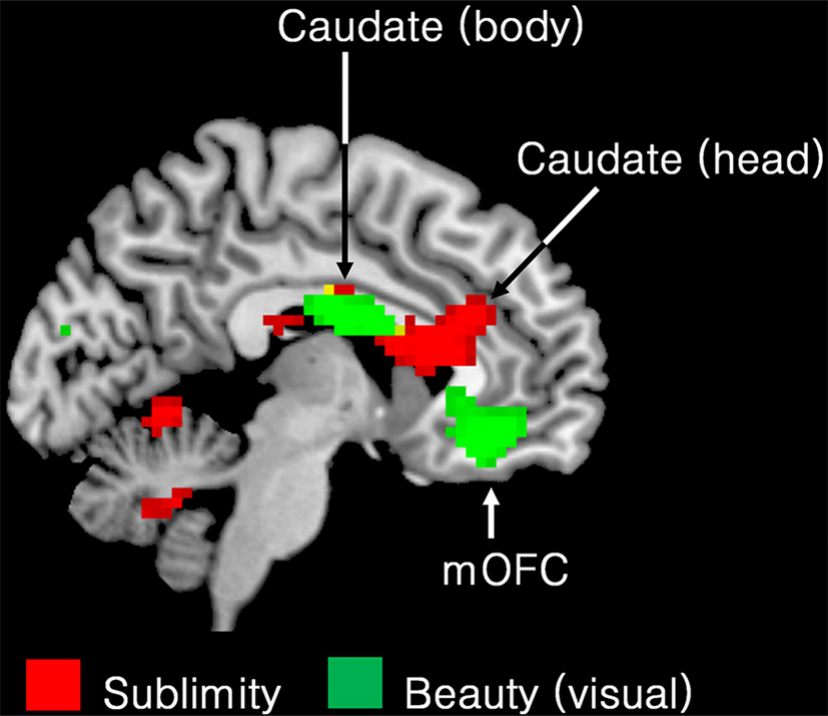
**Superimposed functional maps to show brain areas whose activity correlates with the experience of visual beauty (Ishizu and Zeki, 2011) (green) and the ones in which activity correlates with experience of the sublime (this study, red)**. Activity in the body of the caudate nucleus (−9 −1 25) and in the medial obito-frontal cortex (mOFC) (−6 41 −11) correlates with the experience of visual beauty whereas activity in the head of the caudate, encroaching on anterior cingulate cortex, correlates with experience of the sublime (9 23 16). Both activations are presented using *p* < 0.001 uncorrected threshold.

Another of our aims was to learn about differences between the neural correlates of the experience of the beautiful and the sublime. We therefore compared the regions engaged during the experience of beauty in our previous study (Ishizu and Zeki, 2011) with the regions engaged in the present one, by superimposing the pattern obtained in the former study onto the one obtained in the latter (Figure 4). A glance at this figure shows the profound difference in neural activity that correlates with the two experiences. In particular, field A1 of mOFC, in which activity correlates parametrically with the declared experience of beauty derived from different sources (Ishizu and Zeki, 2011, 2013; Zeki et al., 2014, *inter alia*) was not active in the current study, while other regions, notably the head of the caudate, the putamen, and the posterior hippocampus, in which activity has been recorded as correlating with experiences of pleasure, hate, and memory, respectively, were also active with the experience of sublimity.

Taken together, these results indicate that aesthetic (beauty) experiences and experience of the sublime engage separate and distinct brain systems.

## Discussion

The sublime and its experience occupy a prominent place in philosophical discourse on aesthetics and are often considered as constituting a counterpoint to beauty and its experience. It seems natural, then, that a study of the neural correlates of the experience of beauty should lead ineluctably to a study of its counterpart, the experience of the sublime. Both are topics that are fraught with difficulty and hence our diffidence in approaching the subject and undertaking this study. Any exhaustive study of the sublime would have to consider fields as diverse as rhetorics, tragedy, mathematics, nature, and divinity, all of which have been considered to lead to that sense of elevation [*Erhebung*] that Schopenhauer (1844) wrote of. We tried to render our study more manageable by concentrating on natural scenes, partly because they have occupied such a paramount place in discussions of the sublime and partly because they enabled us to restrict the range of stimuli that we employed in our study. The experience of the sublime has a contradictory element within it, combining the noble with horror. It was indeed the experience of the unfathomable grandeur of the Alps by Dennis (1693), who experienced the sublime there as “mingled with Horrours, and sometimes almost with despair,” the Earl of Shaftesbury (1709) who thought of it as “a wasted mountain … [appearing]… as a noble ruin,” and Joseph Addison (1712) for whom the “Alps… fill the mind with an agreeable kind of horror” that were the trigger for discussions of the sublime among those who brought the subject to prominence in the 18th century.

### “Sublime” as an inclusive term

One of the difficulties in addressing the neurobiology of the sublime is that the word itself unites under a single umbrella many different, even opposed, emotions—“fear,” “horror,” “awe,” “pleasure-pain,” and “pleasure that is only possible by means of a displeasure” (Kant, 1790/1986, §27; 260) are some of the characteristics ascribed to the experience of the sublime. This is compounded by another difficulty, namely that each of these words can, in addition, describe or convey a multitude of feelings. “Fear,” for example, is flexible enough to apply to a variety of emotions and yet imprecise in not specifying the more specific emotion. Fear on viewing a face and the more detached and cognitive fear experienced when viewing natural scenes of immensity differ significantly, yet the word itself subsumes them under the same term. The flexibility is an asset in linguistic discourse but the imprecision is a disadvantage for scientific communication. We here assume that the “sublime” is a distinct cognitive-emotional complex, which involves many components but is distinct from each individually, i.e., that the whole is other than the parts. We emphasize this because a straightforward comparison of our present results with past studies that have explored brain activation with various emotions, such as those of fearful human faces, or of pain, or of pleasure, is neither straightforward nor easy. Moreover, our results did not show any activity in brain areas such as the amygdala and the insula, which have been associated with the experience of fear and threat (Mattavelli et al., 2013; Aube et al., 2014) (although the great majority addressing this question have concentrated on faces) or perceived pain (Cheon et al., 2013; Ellingsen et al., 2013; Favilla et al., 2014), which engages the anterior cingulate cortex, de-activated in our study. Hence the overall pattern of activation in this study is significantly different from the overall activity observed in studies dealing with pain, threat, or fear, each one of which qualifies—at least lexically—as constituting an element in the experience of the sublime. It is therefore perhaps futile to give too rigid an interpretation of the functions of regions that were activated and de-activated in our study in relation to past imaging study that have addressed the individual components (such as fear or surprise) which are subsumed under the term (and experience of) of sublime. This is even more so because, although the regions themselves can be fairly accurately demarcated, a search of the literature reveals that there is no unanimity of views on the overall functions of each of these active regions, beyond the somewhat general statement that they are involved in some kind of emotional experience, with some having cognitive functions as well.

There are further difficulties even within such confines. Both the Earl of Shaftesbury (1709) and Arthur Schopenhauer (1844), among others, regarded the transition from the beautiful to the sublime as graded and gradual. We circumvented this difficulty by asking our subjects to rate the sublimity of the stimuli for themselves and undertook a parametric study of the relationship of the intensity of experience of the sublime to the intensity of cerebral flow. We acknowledge at once that there remains another, presently insurmountable, difficulty even when we restrict ourselves to natural scenes, which is that subjects were asked about their experience of the sublime in images of natural scenes within the confines of a scanner. This inevitably limits the grandeur and depth of the experience and of course limits our conclusions too. Yet, in spite of these restrictions and difficulties, we believe that our results give interesting insights—no more—into the neural mechanisms that are engaged during the experience of the sublime and how they differ from those engaged during the experience of its counterpart, namely beauty.

### Cortical areas in which activity correlates parametrically with the declared intensity of experience of the sublime

#### The posterior hippocampus

The hippocampus has been divided into two broad sub-divisions, an anterior (ventral) one and a posterior one (Fanselow and Dong, 2010 for a review). It has been hypothesized that the posterior division is more engaged by cognitive tasks while the anterior one is more so during emotional experiences. Although a strong cognitive element has also been imputed to experience of the sublime, particularly as regards mathematical sublimity and the role of the imagination (Kant, 1790/1986), characteristics which may properly be called “cognitive,” the great majority of those who have written on the topic of sublimity have also invoked as strong, if not stronger, emotional elements, summarized in terms such as “awe,” “fear,” and “horror.” By this measure, one would have expected some activity in both hypothetical subdivisions of the hippocampus. That the activity was restricted to posterior hippocampus, where activity also correlates with romantic experiences (Bartels and Zeki, 2004; Zeki and Romaya, 2010), leads us to suspect that the proposed subdivisions of the hippocampus along cognitive—emotional lines is inadequate or does not take into account experiences that involve both emotional and cognitive components. At any rate, the activity reported here and elsewhere about positive emotions (Bartels and Zeki, 2004; Zeki and Romaya, 2010) and negative ones (Lang et al., 2009; Pohlack et al., 2012; Shafer and Dolcos, 2012; Poppenk et al., 2013 for a review) does not sit easily within such a subdivision. Interesting to note in this context is a recent report of a difference in the response of anterior and posterior hippocampus to anxiety related to receiving a shock and anxiety related to interpreting the environment as posing a threat (Satpute et al., 2012), which could as well include the threat perceived at a safe distance; the former appears to correlate with activity in anterior hippocampus while the latter with activity in posterior hippocampus, although both can be qualified as emotional experiences.

#### Fusiform gyrus/inferior temporal cortex/ lateral occipital complex

The activity observed in our study extended posteriorly to include visual areas in the inferior temporal cortex and fusiform gyrus, encroaching onto the lateral occipital cortex (LOC), areas that would be expected to be activated by viewing natural scenes. This is consistent with previous results which have shown that the fusiform gyrus and LOC are active when subjects view emotional scenes (Bradley et al., 2003; Sabatinelli et al., 2011). What our results show in addition is that activity in both is parametrically modulated with the declared intensity of experience of the sublime. This has echoes in a previous study which showed that activity in V5, a visual sensory area, is parametrically related to the declared preference for kinetic stimuli (Zeki and Stutters, 2012). Collectively these results thus indicate that activity that is parametrically modulated with subjective, including emotional, experiences is not restricted to “higher” cortical areas but may involve as well visual sensory areas, possibly by feedback from other centers. We also note that we did not observe such parametrically modulated activity in the primary visual cortex (V1) and the adjacent visual areas V2/V3. This suggests that the sublimity modulation in fusiform gyrus and LOC cannot be accounted for by a visual attentional effect.

#### Inferior/middle frontal gyrus

The inferior frontal gyrus activity in this study extended to the middle frontal gyrus, which is not surprising given that previous studies of emotional states have led to activity in both gyri, but the exact role that each gyrus plays remains uncertain. A variety of studies have shown that the inferior frontal gyrus is activated by emotional stimuli (Yamasaki et al., 2002), including emotional imagery and emotional scenes (Sabatinelli et al., 2006, 2011). Interestingly, it has also been found to be active when subjects imagine future events (Viard et al., 2011), hence emphasizing the importance of the imagination in neural terms, just as it has been emphasized in hypothetical terms in past discussions of the sublime.

#### Basal ganglia

The role traditionally ascribed to the basal ganglia, of an important motor center, is undergoing a profound change, to include activity related to emotions as well (see Arsalidou et al., 2013 for a meta-analysis). Previous studies have shown the body of the caudate nucleus to be involved in the experience of beauty while our present one shows that there was parametrically related activity in the head of the right caudate, which is reportedly involved in both emotional and cognitive functions, though more prominently in the latter (Arsalidou et al., 2013). We also observed activity in the right putamen, which also has cognitive and emotional functions of an indeterminate nature, in addition to being involved in emotional motor planning (Monchi et al., 2006; Boecker et al., 2008; Zeki and Romaya, 2008; Ishizu and Zeki, 2013).

#### Cerebellum

As with the basal ganglia, there has been increasing emphasis on the importance of the cerebellum in both emotional and cognitive experiences (Baumann and Mattingley, 2012). An especially interesting example in the present context is the activity in it that correlates with the experience of mathematical beauty (Zeki et al., 2014), an experience that has both emotional and cognitive components and that has been given a special status within the context of the sublime, by both Kant and Schopenhauer. The active area in our study was located in Crus II, and was peak level significant for both hemispheres while it was only cluster level significant in the left hemisphere. Crus II is the cerebellar locus which previous studies have found to be activated with fear (Baumann and Mattingley, 2012).

### Combined emotional and cognitive functions within individual areas

The areas in our study whose activity was parametrically modulated with the intensity of sublime experiences, as well as evidence from previous studies, raises interesting questions as to the extent to which, even areas that have been traditionally regarded as being primarily sensory in function, combine the experiential, sensory and cognitive element with the emotional one.

Taken together with previous results, the results given here lead us to conclude that (i) there is no easy separation between cognitive and emotional components insofar as the functions of these areas are concerned, since all the areas enumerated above have been found in past studies to have been active during tasks belonging to both categories (see also Cromheeke and Mueller, 2014). This is interesting to emphasize in the context of this study because, as our Introduction points out, a review of the history of discussion on the sublime reveals, likewise, that both cognitive and emotional elements of the experience have been emphasized in the past.

The results also lead us to conclude that (ii) the activity in all the above areas, even ones considered to have mainly sensory functions, such as the fusiform gyrus, infero-temporal cortex and LOC, can be modulated by experiences such as that of the sublime, which have a strong emotional component in addition to the cognitive. Whether an emotional modulation of such sensory areas is “top-down” and dependent upon input from “higher” areas or whether it is “bottom-up,” or whether both, is an unresolved question. The parametric modulation of sensory areas with experiences that have an emotional component, as in this study, is interesting to compare with the parametric modulation of activity in an area such as V5 with kinetic stimuli that are preferred but have no strong emotional component (Zeki and Stutters, 2013). This suggests that “bottom-up” processes which are not strictly sensory may also play an important, if not exclusive, role in modulating activity within sensory areas.

### Deactivations

The general comments we make about cortical activations apply as well to the de-activations that we have observed. Of these, the following deserve special comment:

#### Superior frontal gyrus

The most prominently deactivated area, unique to the highest levels of the sublime experience, was the superior frontal gyrus, a zone that was previously found to be de-activated during sensori-motor processing, and interpreted to signify a suppression of self-awareness during such processing (Goldberg et al., 2006). Suppression of self awareness would not be expected during experience of the sublime, which has been written of as leading to an awareness of one's insignificance in relation to the immensity and grandeur of the Nature. This would imply that one is aware of one's existence and insignificance during such experiences.

#### Posterior cingulate cortex/precuneus

Another prominent deactivation, produced by experience of the highest sublimity alone, was found in the PCC/precuneus, which discerns emotional and self-related information (Buckner et al., 2008 for a review). Together with mPFC, which allows for self-reflection and the regulation of emotions (Raichle et al., 2001; Buckner et al., 2008 for a review), and which was deactivated with sublimity in this study, PCC/precuneus have been regarded as key structures of resting-state functional connectivity (or default state network), which has been thought to reflect introspection and self-referential thought (Raichle et al., 2001). Deactivations within those self-referential regions, again, may suggest a linkage between the feeling of awareness of one's insignificance and the experience of sublimity.

#### Cerebellum

The bilateral de-activation observed in Crus I of the cerebellum highlights again the strong cerebellar involvement in emotional and cognitive processing. This particular part of the cerebellum has been previously reported to be activated during the experience of fear (Baumann and Mattingley, 2012), emphasizing well our point above. The term sublime includes the component of fear, but with a very different nuance and emphasis. Hence it may not be surprising to find that areas mediating what is more traditionally understood by fear are suppressed during the experience of fear associated with the sublime, which is an altogether different kind of experience.

Here it is interesting to point out that the deactivation in right cerebellum was significant at both peak and cluster levels while the activation in Crus I (see above) was only significant at peak level. In spite of this difference in statistical significance between activation and de-activation, the proximity and symmetrical distribution of active cerebellar regions, compared to ones that were de-activated, may be of potential interest for future studies exploring similar affective states as this one.

#### Anterior cingulate/medial prefrontal cortex

The above point is made emphatic as well with the observation of a de-activation in the anterior cingulate/medial prefrontal cortex. The latter has been posited to be important during the experience of negative emotions. It is not at all surprising that the experience of the sublime, which has generally been written of as a positive experience, should lead to de-activation in regions which previous studies have implicated in negative experiences (Etkin et al., 2011; Doty et al., 2014).

## Conclusion

We undertook this work to learn whether there is any difference in the neural mechanisms that correlate with two complex experiences that are nevertheless easily distinguishable at the extremes, even if what is beautiful can sometimes also be regarded as sublime and vice versa. We were surprised to find that the neural activity that correlates with experience of the beautiful is very different from that which correlates with experience of the sublime. None of the areas active in studies of visual beauty (Kawabata and Zeki, 2004; Vartanian and Goel, 2004, *inter alia*; reviewed in Ishizu and Zeki, 2011), and not even beauty derived from a highly cognitive source such as mathematics (Zeki et al., 2014) were active in this study, and vice versa.

Burke terminated his book which, among other works, inspired our experiments, thus: “on a review of all that has been said of the effects, as well as the causes of both; it will appear, that the sublime and beautiful are built on principles very different, and that their affections are as different: the great has terror as its basis… the beautiful is founded on mere positive pleasure” (Burke, 1757, 4: XXV). Our experiments, while leaving many details unsettled and many questions unanswered, nevertheless show that the profound distinction between the two experiences emphasized in the past is reflected neurobiologically in the engagement of radically different mechanisms during the two experiences.

### Conflict of interest statement

The authors declare that the research was conducted in the absence of any commercial or financial relationships that could be construed as a potential conflict of interest.
